# Crossmodal interaction of flashes and beeps across time and number follows Bayesian causal inference

**DOI:** 10.3758/s13423-026-02857-z

**Published:** 2026-02-17

**Authors:** Haocheng Zhu, Yiyang Zhang, Ulrik Beierholm, Ladan Shams

**Affiliations:** 1 Department of Psychology, University of California, Los Angeles, CA USA; 2 Department of Psychology, Durham University, Durham, UK; 3 Department of Bioengineering, University of California, Los Angeles, CA USA; 4 Interdepartmental Neuroscience Program, University of California, Los Angeles, CA USA

**Keywords:** Multisensory perception, Bayesian inference, Causal inference, Computational model

## Abstract

Multisensory perception requires the brain to dynamically infer causal relationships between sensory inputs across various dimensions, such as temporal and spatial attributes. Traditionally, Bayesian Causal Inference (BCI) models have generally provided a robust framework for understanding sensory processing in unidimensional settings where stimuli across sensory modalities vary along one dimension such as spatial location, or numerosity (Samad et al., *PloS one*, 10 (2), e0117178, 2015). However, real-world sensory processing involves multidimensional cues, where the alignment of information across multiple dimensions influences whether the brain perceives a unified or segregated source. In an effort to investigate sensory processing in more realistic conditions, this study introduces an expanded BCI model that incorporates multidimensional information, specifically numerosity and temporal discrepancies. Using a modified sound-induced flash illusion (SiFI) paradigm with manipulated audiovisual disparities, we tested the performance of the enhanced BCI model. Results showed that integration probability decreased with increasing temporal discrepancies, and our proposed multidimensional BCI model accurately predicts multisensory perception outcomes under the entire range of stimulus conditions. This multidimensional framework extends the BCI model’s applicability, providing deeper insights into the computational mechanisms underlying multisensory processing and offering a foundation for future quantitative studies on naturalistic sensory processing.

**Supplementary Information:**

The online version contains supplementary material available at 10.3758/s13423-026-02857-z.

## Introduction

Our brains constantly process various types of multisensory signals in daily life, continuously making decisions about whether to integrate or segregate this information (Körding et al., 2007; Shams et al., 2005; Shams & Beierholm, 2010; Stein & Stanford, 2008). For instance, when dining in a restaurant, to better understand what someone is saying, the perceptual system integrates both their voice and lip movements. In this process, it is essential to accurately infer whether the auditory and visual information originate from the same source.

Bayesian Causal Inference (BCI), which is a normative Bayesian model (Körding et al., 2007; Shams & Beierholm, 2010; 2022), has been proposed to account for human multisensory processing. In this normative framework, when processing two or more concurrent sensory inputs, the nervous system relies on uncertain sensory inputs (likelihoods) and prior expectations (prior) to infer the causal structure, with the final cue combination based on the inferred structure. BCI framework has been successfully applied in several multisensory paradigms (see review by Shams & Beierholm, 2022), including the ventriloquist effect (Odegaard et al., 2017; Rohe & Noppeney, 2015; Wozny & Shams, 2011), sound-induced flash illusion (Beierholm et al., 2009; Rohe et al., 2019; Odegaard et al., 2016; Odegaard et al., 2016; Zhu et al., 2024b), size-weight illusion (Peters et al., 2016), rubber hand illusion (Marie Chancel et al., 2022; Samad et al., 2015), and heading perception (Acerbi et al., 2018; Dokka et al., 2019).

Recent modelling work has begun to extend BCI beyond one-dimensional settings. Samad et al. (2015) proposed a two-dimensional Bayesian Causal Inference model to explain the rubber hand illusion, integrating spatial and temporal information from visual, proprioceptive, and tactile cues to infer the causal relationship between the hand and the brush. However, this study used simulations of the model rather than observers’ data fitting. Another study (McGovern et al., 2016) used a two-dimensional spatiotemporal BCI to predict how audiovisual learning would reshape causal-inference judgments. Other studies have also explored additional feature pairings. One study examined the integration of surface features such as roughness and slant in virtual objects and reported that causal inference did not automatically incorporate task-irrelevant dimensions (Badde et al., 2023). Hong et al. (2025) fitted a spatiotemporal BCI model to individual data and reported that observers systematically underestimate auditory spatial and temporal uncertainty. Their model treated spatial and temporal dimensions as independent sources of sensory information, as is standard in multidimensional BCI frameworks.

In everyday life multisensory information often unfolds across multiple dimensions (e.g., spatial, temporal, numerosity) in complex and dynamic ways. This raises a broader question: Can our perceptual system effectively integrate cross-dimensional cues to infer the causal structure of the external world? To explore this question, we propose a normative Bayesian framework that captures how the brain infers causal structures in environments with multidimensional cues. For example, when we watch a person speak, lip movements and speech are usually synchronized; if, however, there was a substantial delay (e.g., 1 s), our perceptual system would likely interpret these signals as arising from different sources rather than a single unified event. The proposed framework not only accommodates these complex facets of multisensory processing but also allows for a more generalized understanding of how laboratory findings may translate to real-world conditions, which inherently involve higher-dimensional and dynamic perceptual signals.

The sound-induced flash illusion (SiFI) (Shams et al., 2000, 2002) is one of the most prominent paradigms used to study multisensory perception (Powers et al., 2009; Rohe et al., 2019; Zhu et al., 2023; see Hirst et al., 2020, for a review). Traditionally, the SiFI has been viewed as a prime example of auditory-dominant perception: because the auditory modality tends to have lower uncertainty in the temporal domain, the visual modality is often drawn into alignment with the perceived auditory events (Shams et al., 2005). To test our multidimensional BCI model, we implemented a modified version of the SiFI paradigm that introduced discrepancies between auditory and visual signals in both the numerosity and the temporal dimensions. By treating these dimensions as separate pieces of evidence, we aimed to determine whether participants could infer the causal structure of multisensory stimuli in a multidimensional context.

Qualitatively, Bayesian Causal Inference would predict that the interaction between the two modalities (and the degree of illusion) would depend on the inference of a common cause. The inference of a common cause would in turn depend on the overall consistency between the sensory inputs (here, auditory and visual) as well as the prior expectation of a common source. The overall consistency between the sensory inputs would depend on consistency across all observed features, here numerosity and timing. Therefore, moderate consistency across both dimensions may result in a similar outcome to a strong consistency in one dimension and weak consistency in another. Because multiple factors influence the inference of the causal structure as well as the inference of the sources (e.g., the number of flashes and beeps), to explore whether the human perceptual system follows Bayesian causal inference, we quantitatively compared the response distributions of each observer with that of the Bayesian Causal Inference (see section *Model comparison*).

Our findings support the idea that the brain’s multisensory processing aligns well with multidimensional BCI, demonstrating that the perceptual system can integrate information from distinct dimensions by accounting for the varying degrees of uncertainty in each modality. These results highlight the brain’s remarkable ability to reconcile complex, multidimensional signals into a coherent representation of reality, offering new insights into the sophisticated computations underlying multisensory perception.

## Methods

### Participants

Twenty-six participants were initially recruited for this study. However, three participants were excluded for failing to meet the inclusion criteria or for performing inadequately in the unisensory conditions of the numerosity judgment task. This resulted in a final sample of 24 participants, aged 17–28 years (mean age = 20.5 years, *SD* = 2.1). All participants were drawn from the UCLA Psychology Department’s subject pool.

The inclusion criteria required participants to have normal or corrected-to-normal vision and hearing. Additionally, they could not have a personal history of epilepsy, photosensitivity, or migraines. All participants were naive to the objectives of the study and provided informed consent through an online form prior to participation. Participants received UCLA Sona credits as part of their academic involvement in the study. The recruitment process, informed consent, and experimental procedures were reviewed and approved by the Institutional Review Board (IRB) at UCLA.

### Apparatus and stimuli

The experiment was conducted on an Apple Mac Mini computer Model A1347 and a CRT monitor with a screen resolution of 1,920 × 1,080 pixels and a refresh rate of 100 Hz. This ensured consistent presentation of visual stimuli and timing accuracy across all trials. The experiment was programmed in MATLAB R2024a using Psychtoolbox, and an Eyelink 1000 eye-tracking system was integrated to ensure participants maintained fixation on a centrally presented cross throughout the task.

Visual stimuli consisted of circular white disks with a diameter of 2° of visual angle, presented for 10 ms at maximum luminance. The disks were positioned approximately 5° below the fixation cross. Auditory stimuli consisted of 10 ms tones at 3.5 kHz. The sound volume was standardized to 80 dB SPL to ensure consistent auditory input across participants. On each trial, participants were presented with one, two, or no flashes paired with one, two, or no beeps. The inter-stimulus interval (ISI) between consecutive beeps or flashes was fixed at 50 ms. The first flash and first beep were presented at one of four possible stimulus-onset asynchronies (SOAs): 0, 150, 300, 500 ms.

### Procedure

The experiment was conducted in a quiet, dark lab environment. Participants sat 55–60 cm from the screen with their chins rested on a chinrest, instructed to maintain fixation on a centrally displayed cross throughout the task. Eye movements were monitored using an Eyelink 1000 device, and participants could only proceed to the next trial when their gaze was fixed on the cross.

**Fig. 1:**
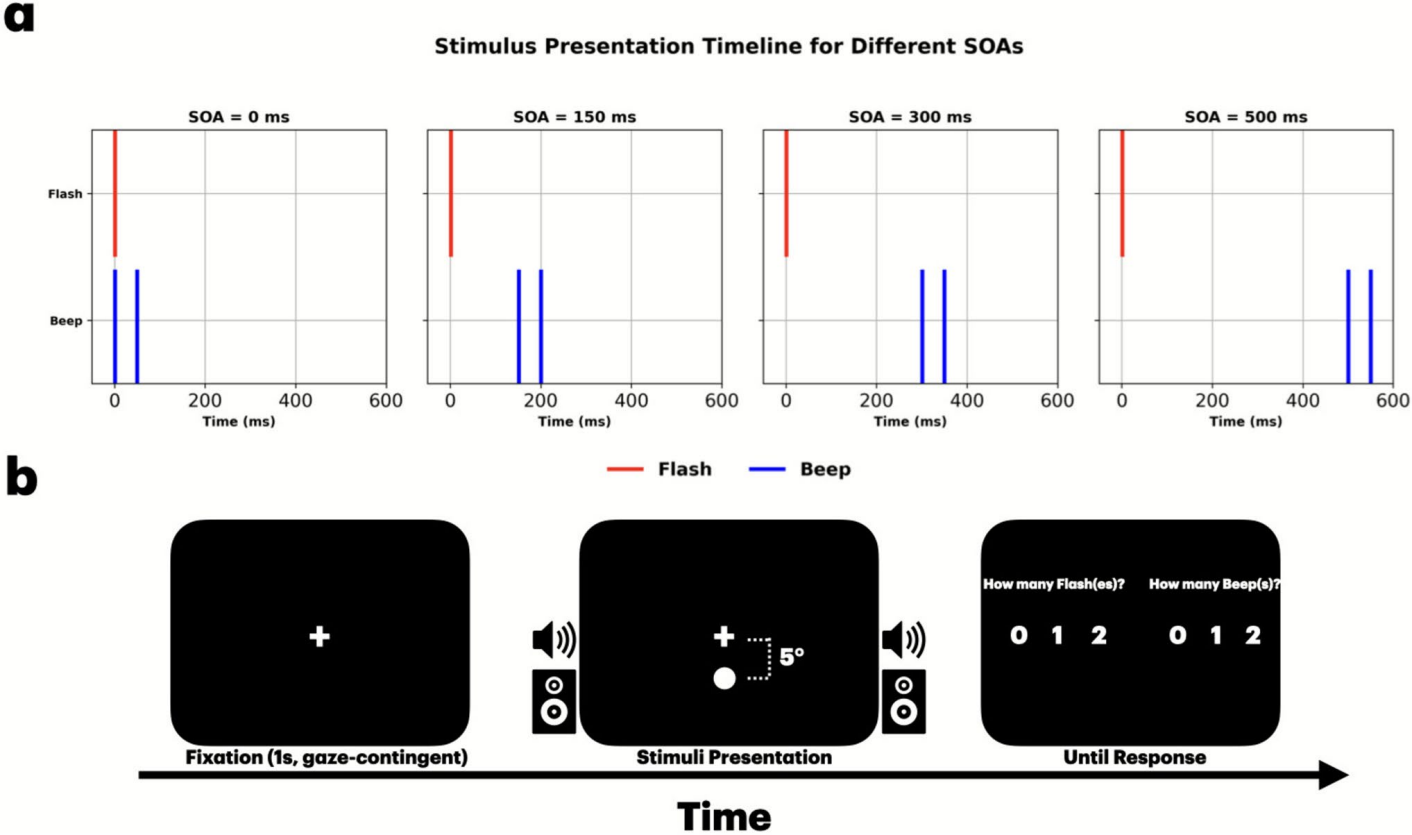
Experimental setup. (**a**) The stimulus timing across the four stimulus-onset asynchronies (SOAs; 0, 150, 300, and 500 ms) is shown for an example flash-beep combination of one flash and two beeps (1F2B). Red and blue lines represent visual (flash) and auditory (beep) stimuli, respectively. (**b**) The trial flow: participants fixate on a central cross, then are presented with auditory-visual stimuli, followed by a prompt for their response, reporting the number of both flashes and beeps on each trial

The experiment included 20 conditions: eight combinations of flash-beep pairings at four Stimulus Onset Asynchronies (SOAs: 0, 150, 300, and 500 ms), and four unisensory conditions (see Fig. 1a). The SOAs represented the temporal delay between the first beep and the first flash, with an SOA of 0 ms indicating simultaneous presentation and an SOA of 500 ms indicating a 500-ms delay. Ten repetitions of each condition, resulting in a total of 200 trials, were presented in a pseudorandom order to each participant. The timing was verified using an oscilloscope.

On each trial, participants reported both the perceived number of flashes and the perceived number of beeps by pressing 0, 1, or 2 (see Fig. 1b). Participants reported their perceived numerosity using a mouse to select the corresponding response option on the response screen, and confirmed their choice by pressing the spacebar. Break screens appeared every 50 trials, allowing participants to rest and reduce fatigue. Including instructions, breaks, and trials, the experiment lasted approximately 30–40 min 2.

**Fig. 2:**
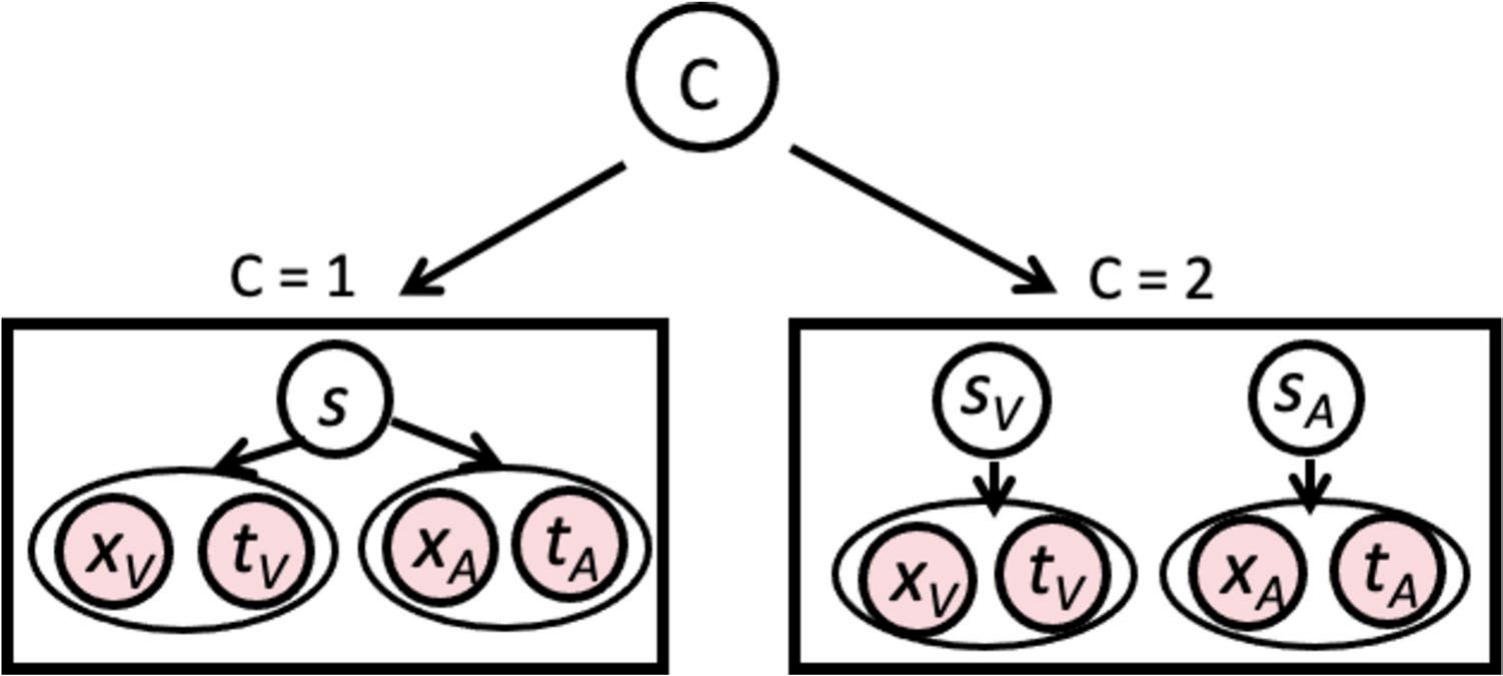
The generative model of multidimensional Bayesian Causal Inference model. Each sensory input (*A* and *V*) has two dimensions: numerosity (*x*) and time (*t*). Both auditory and visual inputs are either caused by the same source (*s*), or by two independent sources (*s*_*V*_ and *s*_*A*_) (Fig. 2)

### Multidimensional Bayesian causal inference model

In principle, the general implementation is based on the BCI model of multisensory perception (Körding et al., 2007; Wozny et al., 2010; Zhu et al., 2024b). To account for the multidimensionality, we extended the classic BCI model to encompass multiple factors. In BCI, prior expectations and current sensory information are used to infer whether sensory stimuli originate from a common cause. Here the sensory input consists of the numerosity (x_V_, x_A_; representing number of flashes and number of beeps, respectively) as well as timing of stimuli (t_V_, t_A_; representing onset of visual stream, and onset of auditory stream, respectively). The inference of the causal structure is computed according to Bayes’ rule (Eq. 1):

1 $$P\left(C|{x}_{V},{x}_{A},{t}_{V},{t}_{A}\right)=\frac{P({x}_{V},{x}_{A},{t}_{V},{t}_{A}| C)P(C)}{P({x}_{V},{x}_{A},{t}_{V},{t}_{A})}$$

During the inference stage of perception, these two hypotheses compete to explain the sensory observations in order to estimate the perceptual variables of interest, for example, the numerosity of the auditory event (*s*_*A*_*)* and the numerosity of the visual event (*s*_*V*_). The underlying causal structure of the stimuli is inferred based on the available sensory evidence and prior knowledge. Each stimulus or event *s* in the world causes a noisy sensation *x*_*i*_ of the event. We use the generative model to simulate experimental trials and subject responses by performing Monte Carlo simulations. Each sensory observation is modelled with a feature-specific likelihood: *p*(*x*_*i*_|*s*) for numerosity and *p*(*t*_*i*_|φ) for time. The numerosity prior is modeled as a Gaussian distribution, p(s) = N(s;μ_p_,σ_p_^2^), where μ_p_ represents the expected numerosity and σ_p_ the uncertainty of this expectation. The temporal prior represents the expected time at which the audiovisual event occurs and is modeled as a Gaussian distribution, p(φ) = N(φ;μ_tp_,σ_tp_^2^), where μ_tp_ denotes the expected event time and σ_tp_ the uncertainty of temporal expectation.

Trial-to-trial variability is introduced by sampling from a normal distribution around the true locations *s*_*A*_ and *s*_*V*_. This simulates the corruption of auditory and visual signals by independent Gaussian noise with standard deviation *σ*_*A*_ and *σ*_*V*_, respectively (Eqs 2 and 3). Here, auditory and visual arrival times (t_V_ and t_A_) are defined relative to a fixed temporal reference point within each trial, namely the onset of the first stimulus in the sequence. All temporal variables therefore represent relative timing within a trial rather than absolute physical time. And the timing of auditory and visual streams are simulated by independent Gaussian noise with standard deviation *σ*_*At*_ and *σ*_*Vt*_ around their true onset times *s*_*At*_ and *s*_*Vt*_, respectively (Eqs 4 and 5).

2 $${x}_{A}\sim N\left({S}_{A}, {\sigma }_{A}^{2}\right)$$

3 $${x}_{V}\sim N\left({S}_{V}, {\sigma }_{V}^{2}\right)$$

4 $${t}_{A}\sim N\left({S}_{At}, {\sigma }_{At}^{2}\right)$$

5 $${t}_{V}\sim N\left({S}_{Vt}, {\sigma }_{Vt}^{2}\right)$$

We assume that number (x_V_ and x_A_) and time (t_V_ and t_A_) are conditionally independent given *C*. The posterior probability of a single cause can be computed by:

6 $$\begin{array}{c}p (C=1|{x}_{V},{x}_{A},{t}_{V},{t}_{A}) = \frac{p({x}_{V},{x}_{A},{t}_{V},{t}_{A}| C = 1)p(C = 1)}{p({x}_{V},{x}_{A},{t}_{V},{t}_{A})}\\ = \frac{ p({x}_{V},{x}_{A}|C = 1)p({t}_{V},{t}_{A}|C = 1)p(C = 1)}{p({x}_{V},{x}_{A})p({t}_{V},{t}_{A})}\\ = \frac{ p({x}_{V},{x}_{A}|C = 1)p({t}_{V},{t}_{A}|C = 1)p(C = 1)}{((p({x}_{V},{x}_{A}|C= 1)p(C= 1)+p({x}_{V},{x}_{A}|C = 2)(1-p(C=1)))(((p({t}_{V},{t}_{A}|C= 1)p(C= 1)+p({t}_{V},{t}_{A}|C = 2)(1-p(C=1)))}\end{array}$$

where *p(C = 1)* is the prior probability of a common cause. The likelihood of experiencing the joint sensations *x*_*A*_ and* x*_*V*_, and the timing of streams* t*_*A*_ and* t*_*V*_ given a causal structure can be found by integrating over the latent variable *s*_*i*_:

7 $$\begin{array}{l}p({x}_{V},{x}_{A}| C = 1) = \int p({x}_{V},{x}_{A}|s)p(s)ds\\ =\int p({x}_{V}|s)p({x}_{A}|s)p(s)ds\end{array}$$

8 $$\begin{array}{l}p\left({t}_{V},{t}_{A}| C=1\right)=\int p\left({t}_{V},{t}_{A}|\varphi \right)p\left(\varphi \right)ds\\ =\int p\left({t}_{V}|\varphi \right)p\left({t}_{A}|\varphi \right)p\left(\varphi \right)d\varphi \end{array}$$

For *p(x*_*V*_*, x*_*A*_*|C= 2)*, we observe that *x*_*V*_ and *x*_*A*_ and *t*_*V*_ and *t*_*A*_ are mutually independent, allowing us to express them as the product of two separate terms:

9 $$\begin{array}{l}p\left({x}_{V},{x}_{A}\right|C=2)=\int \int p({x}_{V},{x}_{A}|{s}_{V},{s}_{A})p({s}_{V},{s}_{A})d{s}_{V}d{s}_{A}\\ =(\int p({x}_{V}|{s}_{V})p({s}_{V})d{s}_{V})(\int p({x}_{A}|{s}_{A})p({s}_{A})d{s}_{A})\\ \begin{array}{l}p({t}_{V},{t}_{A}| C=2)=\int \int p({t}_{V},{t}_{A}|{\varphi }_{V},{\varphi }_{A})p({\varphi }_{V},{\varphi }_{A})d{\varphi }_{V}d{\varphi }_{A}\\ =(\int p({t}_{V}|{\varphi }_{V})p({\varphi }_{V})d{\varphi }_{V})(\int p({t}_{A}|{\varphi }_{A})p\left({\varphi }_{A}\right)d{\varphi }_{A})\end{array}\end{array}$$

The optimal estimation of the sources within each modality is contingent on the causal structure. In the numerosity dimension, if the sensations arise from independent causes, the estimate of *each source* is computed as follows:

10 $$\begin{array}{cc}\widehat s\left(A,C=2\right)=\frac{\frac{x_A}{\sigma_A^2}+\frac{\mu_p}{\sigma_p^2}}{\frac1{\sigma_A^2}+\frac1{\sigma_p^2}},&\widehat s(V,C=2)=\frac{\frac{x_V}{\sigma_V^2}+\frac{\mu_p}{\sigma_p^2}}{\frac1{\sigma_V^2}+\frac1{\sigma_p^2}}\end{array},$$

If the sensations are produced by a common cause, the estimate of *s* is computed as follows:

11 $$\widehat{s}(A,C=1) = \widehat{s}(V,C=1)=\frac{\frac{{x}_{A}}{{\sigma }_{A}^{2}}+\frac{{x}_{V}}{{\sigma }_{V}^{2}}+\frac{{\mu }_{p}}{{\sigma }_{p}^{2}}}{\frac{1}{{\sigma }_{A}^{2}}+\frac{1}{{\sigma }_{V}^{2}}+\frac{1}{{\sigma }_{p}^{2}}}$$

As shown in Eq. 4, the inference regarding the causal structure is probabilistic, leading to uncertainty. The optimal estimates of the sources *sV* and sA depends on the objective of the perceptual system in the specific task at hand. If the objective is to minimize the average error in the estimated source magnitudes – specifically using a sum of squared errors as the cost function – then the optimal approach is model averaging. This method incorporates the estimates from both causal structures, weighting them according to their respective probabilities (Körding et al., 2007; Odegaard & Shams, 2017).

12 $$\begin{array}{c}\widehat{s}A = p(C=1|{x}_{A},{x}_{V},{t}_{V},{t}_{A}) \widehat{s}(A,C=1) + p(C=2|{x}_{A},{x}_{V},{t}_{V},{t}_{A}) \widehat{s}(A,C=2)\\ \widehat{s}V = p(C=1|{x}_{A},{x}_{V},{t}_{V},{t}_{A}) \widehat{s}(V,C=1) +\\ p(C=2|{x}_{A},{x}_{V},{t}_{V},{t}_{A}) \widehat{s}(V,C=2)\end{array}$$

### Model fitting and evaluation

We compared the BCI model with three nested alternatives that have been used in previous multisensory work. (1) Forced-fusion model: the observer always assumes a common cause (C = 1) and combines the auditory and visual likelihoods with a prior over audiovisual numerosity. (2) Maximum Likelihood Estimation (MLE) model: a special case of the forced-fusion model in which the prior is uninformative (flat). In this case, the estimate corresponds to optimal cue combination based solely on the two sensory likelihoods. (3) 1D-BCI model: a reduced version of the two-dimensional (2D) BCI model in which the temporal dimension is ignored, and causal inference is based solely on numerosity information. More details about the alternative models can be found in the Electronic Supplemental Material.

## Results

We examined the perceived number of flashes and beeps as a function of discrepancy between the two modalities both in numerosity and in time. We first analyzed the results from conditions where participants were exposed to varying SOAs in the sound-induced flash illusion (SiFI) paradigm. Subsequently, we assessed how these judgments compare across conditions with different combinations of flashes and beeps. Finally, we compared participants’ responses with predictions of BCI and quantified the goodness of fit of the model.

### Accuracy

While the experiment encompassed 20 unique conditions, our primary focus was on evaluating the effects of SOA on fusion (F2B1) and fission (F1B2) illusions. We first conducted descriptive analyses, followed by statistical evaluations to discern the significance of these effects on perceptual accuracy.

Asterisks indicate significance from Tukey-adjusted pairwise contrasts on the binomial GLMMs. The illusion data were analyzed using generalized linear mixed-effects models (GLMMs) with a binomial link function, implemented in R (lme4 package). Separate models were fitted for the 1F2B and 2F1B conditions, with SOA (0, 150, 300, 500 ms) as a fixed factor and participant as a random intercept. Pairwise contrasts between SOA levels were computed using the emmeans package, with Tukey’s correction applied to control for multiple comparisons.

**Fig. 3:**
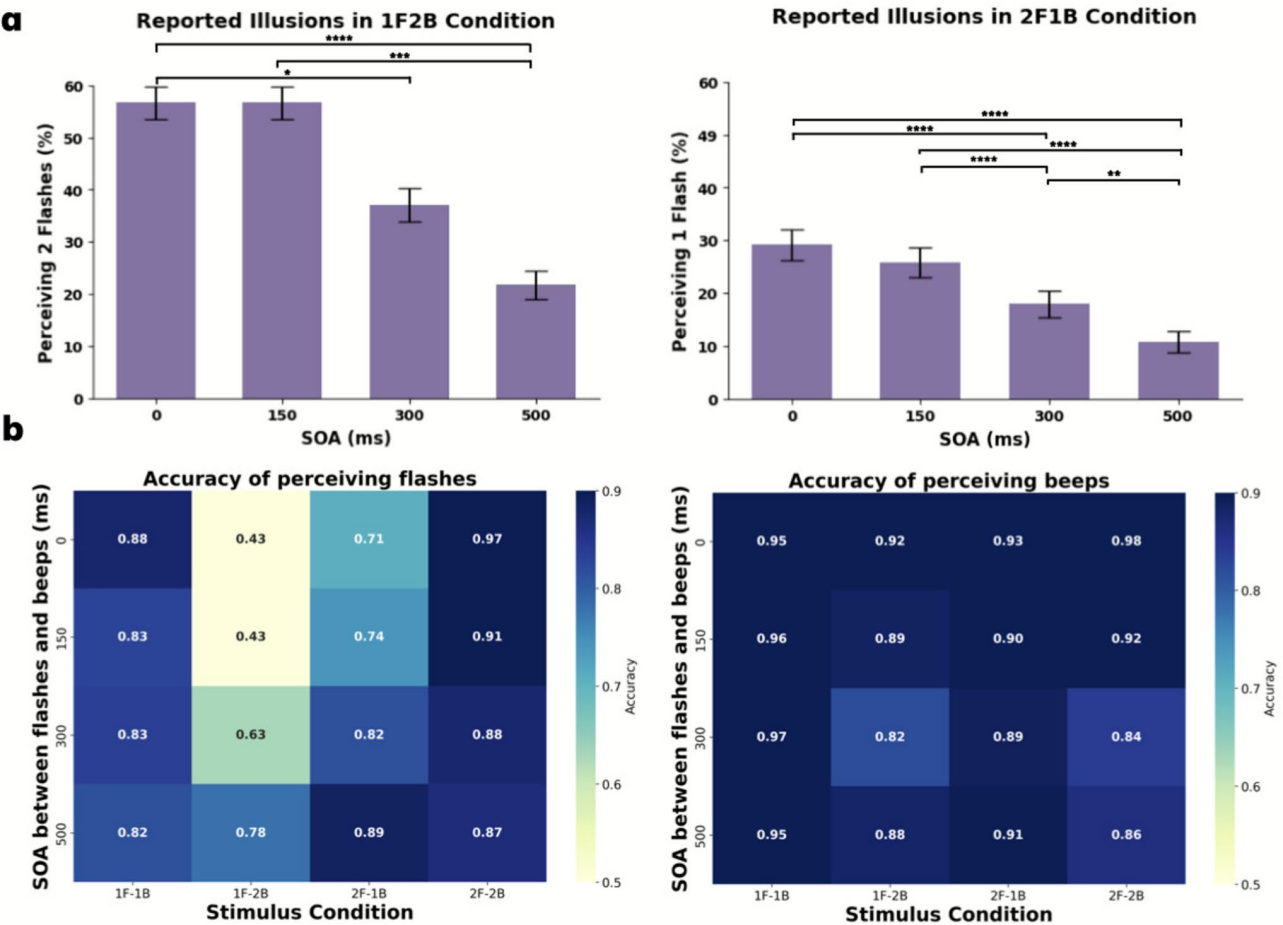
Effect of stimulus-onset asynchrony (SOA) on perception of Sound-induced Flash Illusion. (**a**) Illusion in the 1F2B (left) and 2F1B (right) stimulus conditions across varying SOAs between the stream of flashes and stream of beeps. There is a marked decrease in illusion with increasing SOA for both illusions (****p <.0001). (**b**) The accuracy of flash and beep response across the 20 unique experimental conditions defined by different SOA intervals and flash-beep combinations. In these figures, accuracy is plotted for each flash-beep combination (1F1B, 1F2B, 2F1B, and 2F2B) along the horizontal axis, with SOA (0, 150, 300, and 500 ms) on the vertical axis. The accuracy values are color-coded, where darker shades represent higher accuracy, and lighter shades indicate lower accuracy

As shown in Fig. 3b, in the 1F2B condition, visual accuracy increased substantially from 0.43 at SOA 0 ms to 0.78 at SOA 500 ms. Similarly, in the 2F1B condition, accuracy rose from 0.71 at SOA 0 ms to 0.89 at SOA 500 ms. Auditory accuracy remained relatively high (> 0.8) across all conditions. In the 1F2B condition, accuracy ranges from 0.82 at SOA 300 ms to 0.92 at SOA 0 ms. In the 2F1B condition, accuracy ranges from 0.89 at SOA 300 ms to 0.93 at SOA 0 ms, indicating stable perception of auditory stimuli under different SOAs. Note that in our paradigm, SOA is defined as the onset difference between the first flash and the first beep, which differs from many “classic” SIFI studies that use SOA definitions tied to other event pairs or the overall stream.

The effect of temporal asynchrony on perceptual accuracy was assessed using logistic regression with a binomial distribution, modeling accuracy as a binary outcome. Analyses were conducted separately for congruent and incongruent audiovisual trials, given that multisensory benefits are known to occur primarily under congruent conditions, whereas perceptual illusions occur in incongruent trials.

In the congruent condition, increasing SOA significantly reduced flash accuracy, (*b* = −0.00144, *SE* = 0.00037, *z* = −3.86, *p* <.001). The intercept corresponds to a model-predicted accuracy of approximately 90.8% at SOA = 0 ms, decreasing to 82.9% at SOA = 500 ms. These results suggest that temporal misalignment diminishes multisensory facilitation of visual accuracy. Beep localization showed a similar pattern, with a significant negative effect of SOA (*b* = −0.00185, *SE* = 0.00049, *z* = −3.80, *p* <.001). The model intercept corresponds to a predicted accuracy of 95.5% at SOA = 0 ms, decreasing to 89.5% at SOA = 500 ms.

In the incongruent condition, flash accuracy increased significantly with SOA (*b* = 0.00272, *SE* = 0.00028, *z* = 9.57, *p* <.001), indicating that greater temporal separation reduces cross-modal interaction. The intercept was marginally significant (*p* =.053), consistent with numerically near-chance performance (~53.7%) at SOA = 0 ms. In contrast, SOA had no significant effect on beep accuracy, *b* = −0.00062, *p* =.117, with a consistently high baseline accuracy (~90.5%), indicating the auditory report remained robust despite temporal misalignment.

In summary, temporal asynchrony modulates perceptual accuracy in a condition-specific manner: in congruent trials, short SOAs enhance multisensory integration, while in incongruent trials, longer SOAs improve flash accuracy by reducing cross-modal interaction. Beep accuracy was more resilient overall, particularly under numerically incongruent conditions.

### Modeling results

#### BCI Model

**Fig. 4:**
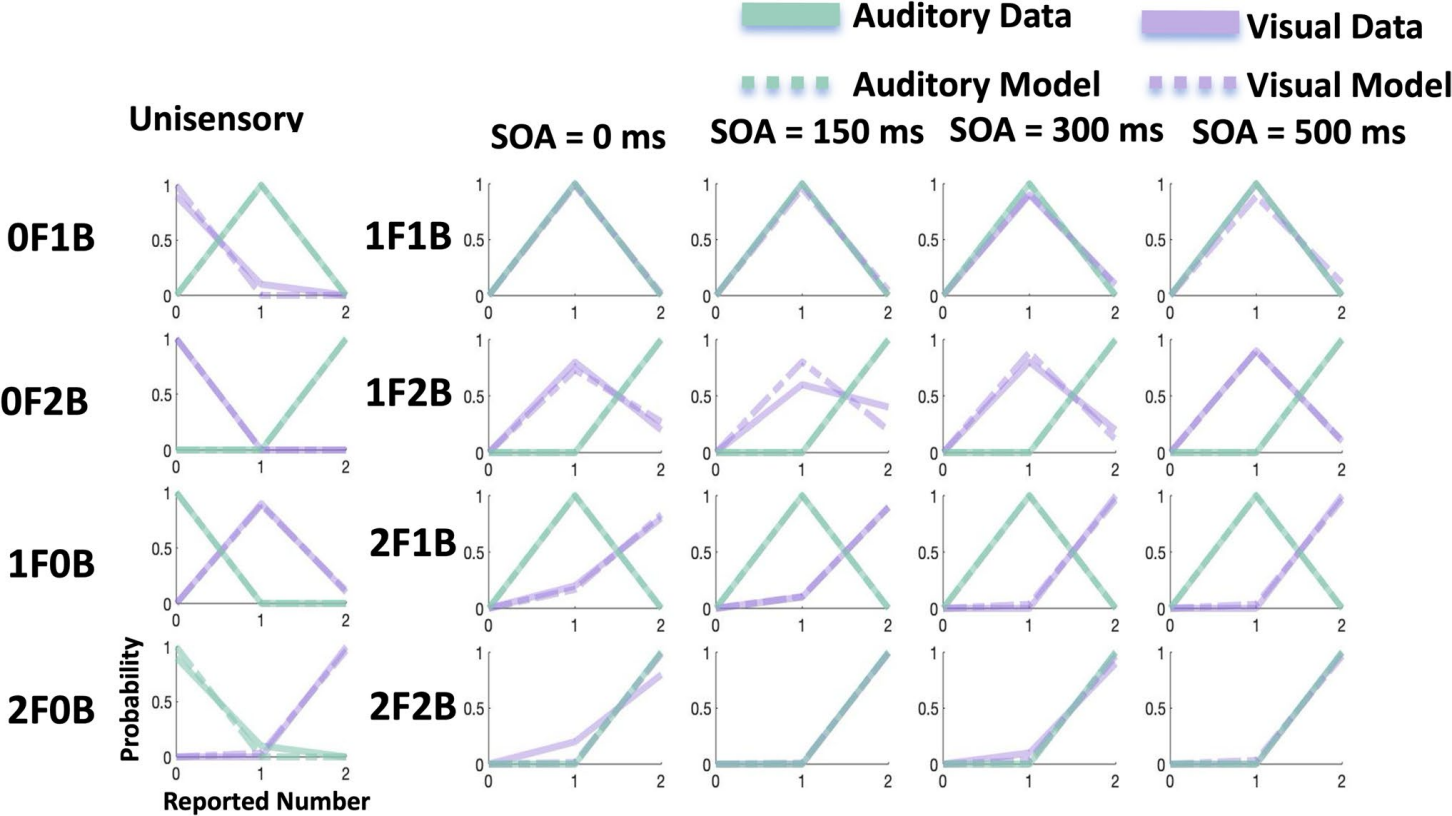
The model fitting results for a representative subject. Solid purple lines correspond to the visual response data, and green lines correspond to the auditory response data. The dashed purple lines represent the model predictions for vision, and the dashed green lines represent the model predictions for audition

Because we lacked participant report data on the timing of visual and auditory stimuli, we chose to fix the corresponding parameters (σ_*At*_ = 40, σ_V*t*_ = 60, μ_tp_ = 100, and σ_tp_ = 500) based on prior research (Zhu et al., 2024b). This additional restriction simplifies the model and makes the fitting procedure more reliable. Using only five free parameters to fit 200 data points, the multidimensional BCI model demonstrated a strong ability to account for the data, achieving a mean *R*^2^ of 0.93 across 24 subjects. To assess the model’s performance, we estimated the parameters for each subject individually using a maximum-likelihood procedure (Table 1) (Fig. 4.)

**Table 1:** Sample mean ± SE parameter estimates across participants

*N* = 24	Likelihood			Prior	
	*σ*_*V*_	*σ*_*A*_	*p*_*commom*_	*σ*_*p*_	*μ*_*p*_
	0.63 ± 0.07	0.33 ± 0.003	0.62 ± 0.06	1.33 ± 0.24	1.43 ± 0.12

#### Model comparison

To test the competing hypothesis that observers base causal inference solely on numerosity, we also fitted a one-dimensional (1D) BCI model that ignores temporal information. Mathematically, this model retains the numerosity likelihoods of Eq. 6 but excludes all temporal terms. Model comparison based on the Bayesian Information Criterion (BIC) revealed that the 2D-BCI model (BIC = 294.56 ± 28.58) provided a substantially better fit than the 1D-BCI (332.43 ± 28.42), forced-fusion (378.12 ± 20.29), and MLE (383.21 ± 20.48) models. Bonferroni-corrected pairwise t-tests confirmed that the 2D-BCI model significantly outperformed all alternatives, 1D-BCI (*t*(22) = –24.74, *p* = 4.52 × 10⁻^17^, *d* = –5.16), MLE (*t*(22) = –7.35, *p* = 7.02 × 10⁻^7^, *d* = –1.53), and forced-fusion (*t*(22) = –8.05, *p* = 1.60 × 10⁻^7^, *d* = –1.68). Detailed statistical results and visualizations of BIC differences are provided in the Electronic Supplemental Material.

## Discussion

In this study, we presented visual and auditory stimuli with varying numerical and temporal discrepancies to investigate how the human brain computes multisensory integration. Our results indicate that the nervous system infers the causal structure underlying sensory inputs by integrating information across multiple dimensions, ultimately deciding whether to combine or segregate these signals in a probabilistic manner.

Multisensory perception has been widely studied, with research showing its benefits for memory (Matusz et al., 2017; Murray et al., 2022; Quak et al., 2015), perceptual learning (Shams & Seitz, 2008), and decision-making (Stein et al., 2020). In our study, increasing temporal discrepancies led to a reduction in the strength of multisensory illusions in incongruent conditions and a diminished multisensory benefit in congruent conditions, indicating a weakening of integration. While these findings highlight the importance of temporal synchrony, they do not imply that integration is determined solely by a fixed temporal binding window. In fact, the BCI model predicts that integration is driven by overall consistency across multiple sensory dimensions. For example, if stimuli are highly consistent in three out of four dimensions (such as spatial location, numerosity, and intensity), even a weak temporal consistency might be sufficient to promote integration. It is the cumulative consistency that ultimately guides the perceptual system’s decision to fuse or segregate sensory inputs.

Despite these advances, quantifying sensory integration and segregation remains a challenge. The BCI model provides a robust framework by suggesting that the nervous system combines prior knowledge with current sensory evidence to infer the underlying causal structure (Körding et al., 2007; Odegaard et al., 2015, 2017; Rohe et al., 2019; Shams & Beierholm, 2010, 2022; Wozny et al., 2010). This framework allows for direct predictions about whether inputs should be integrated or segregated. Behavioral studies have extensively validated the BCI model (Chancel & Ehrsson, 2023; Dokka et al., 2019; Körding et al., 2007; Odegaard et al., 2017; Peters et al., 2016; Wozny et al., 2010; see Shams & Beierholm., 2022, for a review), and recent neuroimaging findings further support the notion that neural circuits perform similar causal inference processes (Cao et al., 2019; Fang et al., 2019; Jones & Noppeney, 2024; Rohe & Noppeney, 2015; Rohe et al., 2019). However, as experimental paradigms evolve to better mimic the complexity of real-world sensory environments, there is an increasing need to extend the BCI framework to account for multidimensional stimuli. In response to this challenge, Zhu et al. (2024b) proposed a modified BCI model. Building on their work, our study tested whether this multidimensional approach could generalize across a broader range of conditions, and can quantitatively account for human perceptual data.

Our findings demonstrate that the multidimensional BCI model robustly predicts multisensory processing, capturing complex interactions with high accuracy. We propose that the brain employs parallel probabilistic processes to integrate information across various dimensions, such as temporal and numerical cues, to infer the causal structure of sensory events. Notably, a discrepancy in any one dimension (e.g., a substantial temporal mismatch) can override numerical consistency, prompting the system to treat the stimuli as originating from separate sources. Also, the near-ceiling accuracy for the beep stream merely reflects its intrinsically higher temporal precision (*σ*_*A*_ = 0.33 vs. *σ*_*V*_ = 0.63) and is explicitly captured by the model, so the predicted auditory dominance at short SOAs, and its disappearance at larger offsets, arises naturally rather than obscuring subtler cross-modal interactions. The present study, together with Samad et al. (2015), Hong et al. (2025) and McGovern et al. (2016), contributes to a growing body of work extending the BCI framework to multidimensional perceptual domains. All these models share the core goal of describing how temporal information constrains multisensory integration, yet differ in their specific formulations. Hong and colleagues modeled spatial and temporal information, defining the temporal variable as the SOA between auditory and visual events, which effectively captures how temporal discrepancies influence causal inference. In contrast, our model focuses on numerosity and temporal alignment, representing the arrival times of each modality (tₐ and tᵥ) as independent latent variables (Zhu et al., 2024a, 2024b). In the present study, all temporal parameters were fixed a priori, such that the temporal component does not introduce additional degrees of freedom or influence model comparison outcomes. Under these constraints, specifying the temporal process either via relative SOAs or via individual auditory and visual arrival times is mathematically equivalent. We adopt the arrival-time formulation to maintain a unified model structure across temporal and numerosity dimensions. Estimating flexible arrival-time parameters would be best supported by additional data from dedicated temporal tasks (e.g., simultaneity judgment). This general formulation may also be extended to paradigms involving absolute timing or more complex temporal structures, and, with our model and code openly available, we encourage others to build upon and extend these models in future work.

While our model adopts Gaussian likelihoods to describe internal estimates of numerosity and temporal intervals, we acknowledge that these assumptions may appear at odds with the discrete or strictly positive nature of the corresponding physical variables. However, theoretical and empirical evidence from neurophysiology and behavior support the use of Gaussian approximations at the internal sensory level. Neuronal populations in the intraparietal cortex encode numerosity on a compressed, analog scale using broad log-Gaussian tuning curves (Jeong et al., 2025; Nieder & Merten, 2007), and behavioral data across species converge on a logarithmically compressed “mental number line” (Dehaene, 1992). Within this analog representation, internal sensory noise is well captured by a Gaussian distribution, consistent with the structure of nearly all existing BCI models (e.g., Shams et al., 2005; Wozny et al., 2008). A similar rationale holds for temporal estimation: internal timing mechanisms integrate numerous small, additive noise sources, such as conduction jitter and synaptic variability, resulting in approximately Gaussian estimation errors by virtue of the central limit theorem (Gibbon, 1977; Ivry & Schlerf, 2008). This assumption is further supported by psychophysical findings, which show that interval reproduction errors are symmetric and Gaussian-like when scaled by elapsed duration (Jazayeri & Shadlen, 2010), and that Bayesian models with Gaussian noise explain hallmark effects such as central tendency and the variance–time tradeoff (Petzschner et al., 2015). Importantly, our findings remain robust when a log-normal likelihood is substituted for numerosity, as shown in our re-analysis (please see Electronic Supplemental Materials). The resulting change in model fit (ΔBIC = +0.06) is negligible, indicating that our core conclusions are not sensitive to whether the internal likelihood is modeled using a Gaussian or a strictly positive alternative.

One limitation of this study is that the temporal-uncertainty parameters were fixed a priori, rather than estimated from the present data, because participants reported only numerosity. While this choice prevents over-fitting in an under-constrained parameter space, it also limits the model’s flexibility and can be revisited in future work by collecting explicit temporal judgements from the same observers. Moreover, the sample in this study was demographically narrow (24 university students, 19–32 years old), so broader populations remain to be tested. The stimuli used in this study consisted of simple flashes and beeps. While these stimuli offer high experimental control but they are limited in ecological validity; confirming the effect with naturalistic audiovisual events is an important next step. Also, the numerosity range in the present paradigm was restricted to 0–2, which provides a minimal test of cross-modal integration. Future studies could extend this framework to higher numerosities to examine whether similar causal-inference dynamics generalize across more complex quantitative representations.

While traditional multisensory studies often involve manipulating only one dimension of the perceptual variables, real-world sensory processing is inherently multidimensional. Our findings suggest that perceptual inference is more flexible and probabilistic than previously assumed, extending beyond rigid cue combination rules. This has practical applications in fields such as human-computer interaction, virtual reality, and clinical neuroscience. For example, understanding how individuals infer causal relationships in noisy environments can improve the design of assistive technologies for sensory impairments. Finally, our work opens new avenues for future quantitative investigations of multisensory integration (Zhu et al., 2024a). By systematically manipulating temporal discrepancies and other sensory features, future studies can further elucidate the mechanisms that define the temporal binding window and govern the integration and segregation processes in naturalistic settings.

## Conclusion

In the present study, we proposed a novel framework for the BCI model that incorporates information from different dimensions of perceptual stimuli. Using a behavioral approach, we manipulated the numerical and temporal discrepancies between audiovisual stimuli to evaluate the model. Our findings indicate that the multidimensional BCI model provides highly accurate predictions for multisensory perception under complex conditions. This study further supports the notion that the brain performs Bayesian causal inference during multisensory processing. Moreover, our newly proposed BCI framework offers substantial potential for enabling more quantitative investigations into complex multisensory research.

## Supplementary Information

Below is the link to the electronic supplementary material.

Supplementary file1 (DOCX 924 KB)

## Data Availability

We shared our data and analysis at https://osf.io/bqt9e/?view_only=e5a068fea53a4970879c54762b67a346

## Code availability

We shared our codes at https://osf.io/bqt9e/?view_only=e5a068fea53a4970879c54762b67a346
