# Supplementary material for: Crossmodal interaction of flashes and beeps across time and number follows Bayesian causal inference
Source: Psychon Bull Rev. 2026 Feb 17;33(3):58. doi: 10.3758/s13423-026-02857-z (PMC12913353; doi:10.3758/s13423-026-02857-z)
Supplement: Supplementary file 1 — Supplementary file1 (DOCX 924 KB) [file 13423_2026_2857_MOESM1_ESM.docx]

**Supplemental Materials: Crossmodal Interaction of Flashes and Beeps Across Time and Number Follows Bayesian Causal Inference**

Haocheng Zhu, Yiyang Zhang, Ulrik Beierholm, Ladan Shams

**Supplemental Materials**

**Supplemental Methods**

**Procedure**

At the beginning of each block, the fixation instruction “Please fix your eyes on the cross at ALL times.” was displayed until the participant pressed the Space key to begin. Afterward, the program waited for 2 seconds before the fixation period began. During each trial, a white fixation cross appeared at the center of a black screen. The fixation period required the participant to maintain continuous gaze within a 100 × 100 pixel window centered on the cross for at least 1000 ms. Therefore, the total fixation duration before stimulus onset was at least approximately 1.0 second, plus any extra time needed for the participant to achieve and maintain fixation.

Following the fixation period, the visual and auditory stimuli were presented. The timing between the onset of the first auditory stimulus and the first visual stimulus was governed by a fixed stimulus onset asynchrony (SOA), selected from the set {0, 150, 300, 500 ms}. Thus, the auditory onset was fixed relative to the first visual stimulus for each trial. After the final stimulus in a trial, the fixation cross remained visible for an additional 1 second, after which the response screen appeared for participant input. Once participants submitted their responses by pressing the spacebar, a 1-second inter-trial interval followed before the next trial began.

**Model Log-Likelihood Function**

For parameter estimation, the 2D-BCI model was fitted by maximizing the log-likelihood of the observed response proportions across conditions. A small lapse rate ε was included to prevent zero probabilities and to account for occasional random guesses. The negative log-likelihood minimized during fitting was:


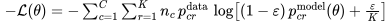
(Eq. S1)

where p_cr_^data^​ are the empirical response proportions in condition c, n_c_​ the number of trials in that condition, K the number of response categories, and p_cr_^model^(θ) the model-predicted probabilities (derived from the posterior after binning).

**Individual-Level Model Fitting and Group-Level Visualization**

To examine how well the two-dimensional Bayesian Causal Inference (2D-BCI) model captures participants’ responses across all experimental conditions, we performed individual-level model fitting for each participant. Model parameters were estimated separately for each observer by minimizing the negative log-likelihood between the model predictions and the empirical response probabilities.

The fitted model predictions and observed data were then averaged across participants. For visualization, we plotted the group mean **(±** SEM**)** of the reported numerosity probabilities for each condition and modality. This allows direct comparison between empirical data (solid lines) and model predictions (dashed lines) for both auditory and visual reports across unisensory and multisensory (SOA) conditions. As shown in Figure S1, the 2D-BCI model provides an excellent fit to the behavioral data across all temporal offsets, successfully reproducing both the fusion (e.g., 1F2B) and fission (e.g., 2F1B) illusion patterns as well as unisensory baselines.


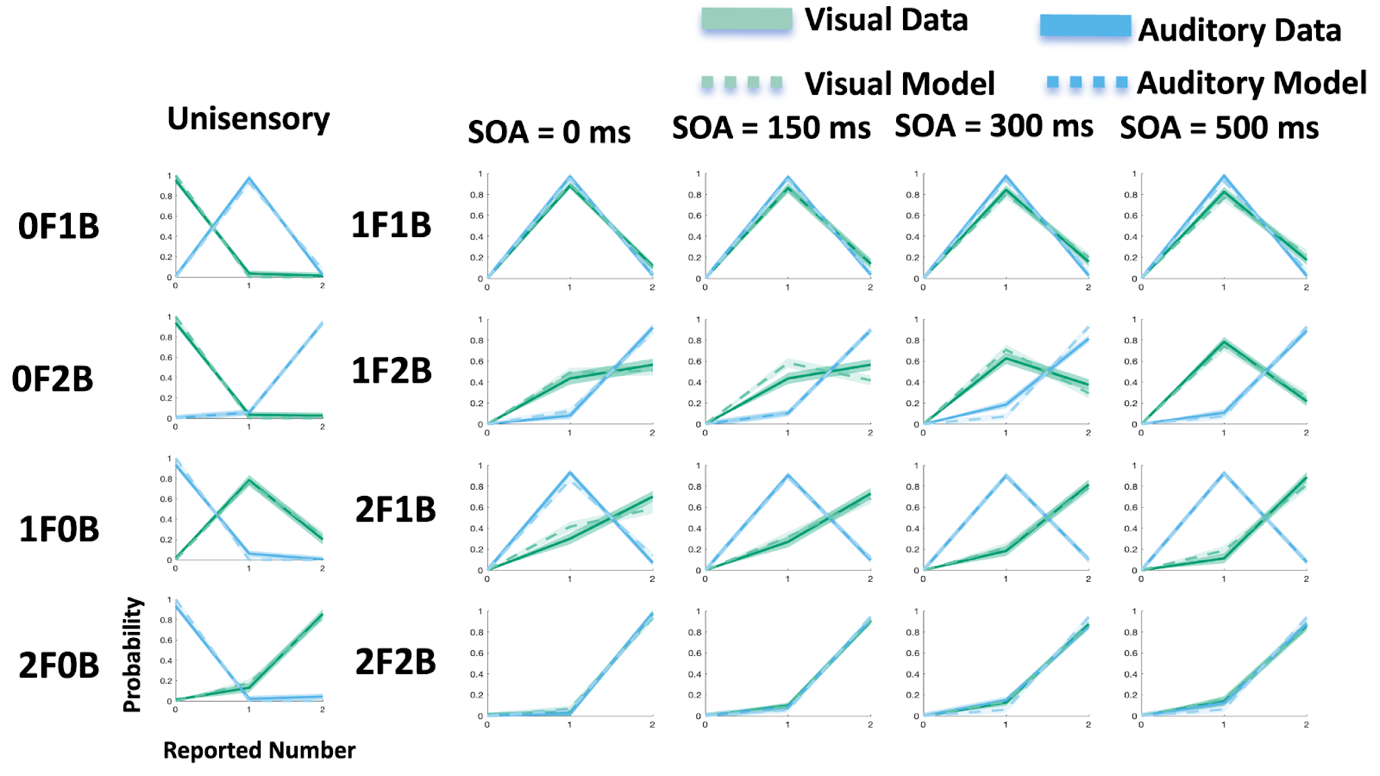


**Figure S1. Individual-level model fitting and group-level visualization.**

Empirical and model-predicted response probabilities are shown for visual (green) and auditory (blue) reports across all experimental conditions. Solid lines represent observed data (mean ± SEM across 23 participants), and dashed lines represent corresponding 2D-BCI model predictions. Columns indicate different audiovisual stimulus onset asynchronies (SOAs), and rows represent different flash–beep combinations. The 2D-BCI model captures both unisensory baselines and multisensory illusion patterns across SOAs, closely matching participants’ empirical response profiles.

**Model Comparison**

The **Bayesian forced-fusion** model operates under the assumption that all sensory inputs originate from a single, common source. It infers this source by combining sensory evidence, represented through likelihood functions with prior expectations. Essentially, this model represents a constrained version of Bayesian causal inference, where the probability of a shared origin for the inputs is fixed at 1 (Eq. S1). When both the likelihood and prior distributions are Gaussian, and the estimation follows a maximum a posteriori (MAP) approach, the inferred source is determined as a weighted sum of the two sensory signals (*x_A_* and *x_V_*) along with the prior. The contribution of each component to the final estimate is governed by its precision (or reliability), which is mathematically characterized as the inverse of its variance.

$\hat{s} =\frac{\frac{x_{A}}{\sigma_{A}^{2}}+\frac{x_{V}}{\sigma_{V}^{2}}+\frac{\mu_{p}}{\sigma_{p}^{2}}}{\frac{1}{\sigma_{A}^{2}}+\frac{1}{\sigma_{V}^{2}}+\frac{1}{\sigma_{p}^{2}}}$                                   (Eq. S2)

The **Maximum Likelihood Estimation** (MLE) model also assumes that all signals originate from a common source, but estimates this source by maximizing the overall likelihood of the combined sensory inputs. In this framework, each signal contributes to the final estimate based on its relative precision, resulting in a weighted average of the observations (Eq. S2). Unlike the Bayesian forced-fusion model, the MLE approach does not incorporate prior information. Instead, it formulates the fusion process purely as an optimization task, relying solely on the likelihoods (or reliabilities) of the signals. This method ensures that the final estimate corresponds to the most probable percept under the assumption of a single-source origin.

$\hat{s} =\frac{\frac{x_{A}}{\sigma_{A}^{2}}+\frac{\mu_{p}}{\sigma_{V}^{2}}}{\frac{1}{\sigma_{A}^{2}}+\frac{1}{\sigma_{V}^{2}}}$(Eq.S3)

**The uni-dimensional (numerosity) BCI model** performs computations solely on the numerosity signals and ignores temporal information. The posterior probability that the two sensory observations arise from a common cause is

$p (C=1|x_{V},x_{A}) = \frac{p(x_{V},x_{A}| C = 1)p(C = 1)}{p(x_{V},x_{A})}$ (Eq. S4)

To quantitatively compare the Bayesian causal inference (BCI) model with the forced-fusion, Maximum Likelihood Estimation (MLE) models and 1-D BCI model, we computed the Bayesian Information Criterion (BIC) for each. Our analyses indicate that the 2D-BCI model achieves a BIC of 294.56 ± 28.58, while the forced-fusion, MLE models and 1D-BCI yield BIC values of 378.12 ± 20.29, 383.21 ± 20.48, and 332.43  ± 28.42 respectively. To statistically assess whether there are significant differences in model fits, we conducted a one-way analysis of variance (ANOVA) on the BIC values across the three models. The ANOVA revealed a significant main effect of model type on BIC values (*F*(3, 30) = 2.84, *p* = .0422), indicating that at least one model significantly differs from the others in terms of fit.

To further explore these differences, we conducted Bonferroni-corrected pairwise tests and confirmed that the 2D-BCI model significantly outperformed all alternatives (Figure S2), 1D-BCI (t(22) = –24.74, p = 4.52 × 10⁻^17^, d = –5.16), MLE (t(22) = –7.35, p = 7.02 × 10⁻^7^, d = –1.53), and forced-fusion (t(22) = –8.05, p = 1.60 × 10⁻^7^, d = –1.68).


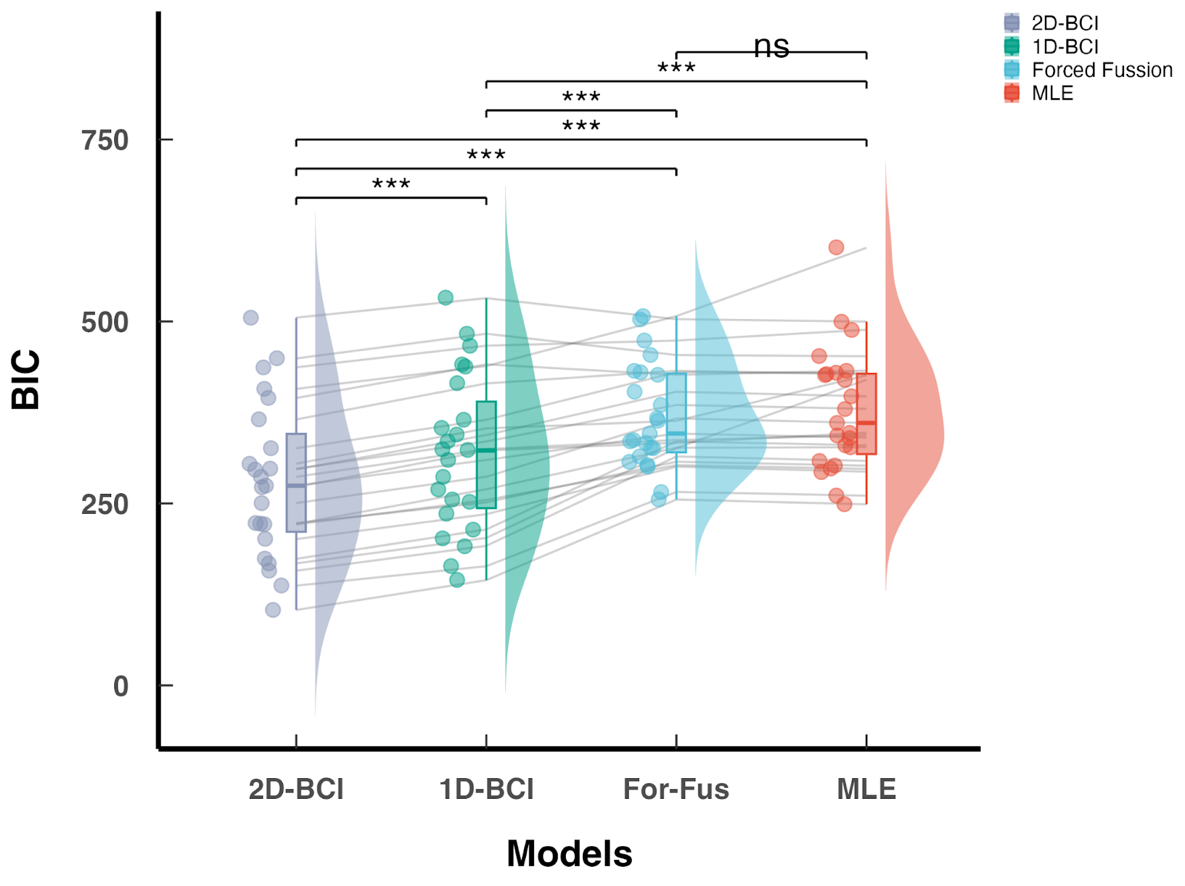


**Figure S2. Model comparison using Bayesian Information Criterion (BIC).**

Individual participants’ BIC values are shown for the four competing models: the full two-dimensional Bayesian Causal Inference model (2D-BCI), a reduced one-dimensional variant ignoring temporal information (1D-BCI), a forced-fusion model assuming a single common cause (For-Fus), and a Maximum Likelihood Estimation model (MLE) without priors. Each dot represents one participant, connected by gray lines to indicate within-subject comparisons. Kernel density estimates and overlaid boxplots show the group-level distribution of BIC values. Lower BIC values indicate better model fit. Horizontal bars denote results of Bonferroni-corrected pairwise comparisons (***p < .001; ns, not significant).

**Numerosity Log-Normal Control Analysis**

The main model assumes Gaussian sensory noise on the internal magnitude representation of numerosity. Although this is the standard choice in BCI studies (Wozny et al., 2008; Körding et al., 2007; Shams et al., 2005) and is motivated by single-unit and fMRI evidence for (log-) Gaussian tuning along the mental number line (Nieder, 2004; Jeong et al., 2025), a Gaussian does assign negligible but non-zero probability to negative values. To demonstrate that our conclusions do not hinge on the support of the likelihood, we repeated the entire fitting procedure with **log-normal** sensory likelihoods while leaving every other assumption unchanged.

Original Eqns 2–3 assumed independent Gaussian noise on the visual and auditory magnitude estimates. They now read:

x_V_​∼LogNormal(μ_V_=log(s),σ^2^_V_​)

x_A​_∼LogNormal(μ_A_=log(s),σ^2^_A_​) (Eq. S5)

where the variance parameters σV2​,σA2​ are now defined in log space.

With a log-normal prior P(ln *s*), the joint likelihood becomes:


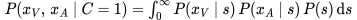
(Eq. S6)

Completing the square shows that this product is itself proportional to a Gaussian whose precision is the sum of the individual precisions,and whose mean is the precision-weighted average:


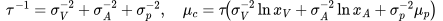
(Eq. S7)

Evaluating the remaining Gaussian integral yields the closed-form likelihood:


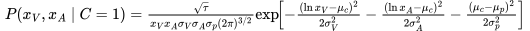
(Eq. S8)

The new model required no extra parameters. For the 120 data points the Bayesian Information Criterion changed from 294.56 (Gaussian) to 294.62 (log-normal), ΔBIC = +0.06. A ΔBIC of 0.06 is far below the conventional threshold (ΔBIC > 6) for substantial evidence. Thus the Gaussian and log-normal formulations are empirically indistinguishable; all main-text conclusions are unaffected.

**Parameter recovery**

To demonstrate that the five free parameters of the two-dimensional BCI model are identifiable, and that our model can recover the true generating model, we performed a full simulation-and-refit exercise.

We first generated one hundred synthetic data sets. For each simulation a parameter vector Θ={pcommon​,σ_1_​,σ_2​_,σ_p_​,μ_p_​} was sampled uniformly from the same ranges used as priors in the empirical fits. All one hundred data sets were then refitted with the maximum-likelihood pipeline described in the Methods section of the main text, using the same search grids, starting seeds, and convergence criteria. Figure S3 plots the recovered parameter estimates against their ground-truth values. The recovery is excellent: Slopes are close to unity in every panel, indicating negligible systematic bias. The modestly lower R^2^ for μ_p_​ is expected because that parameter is least directly constrained by the present stimulus range.


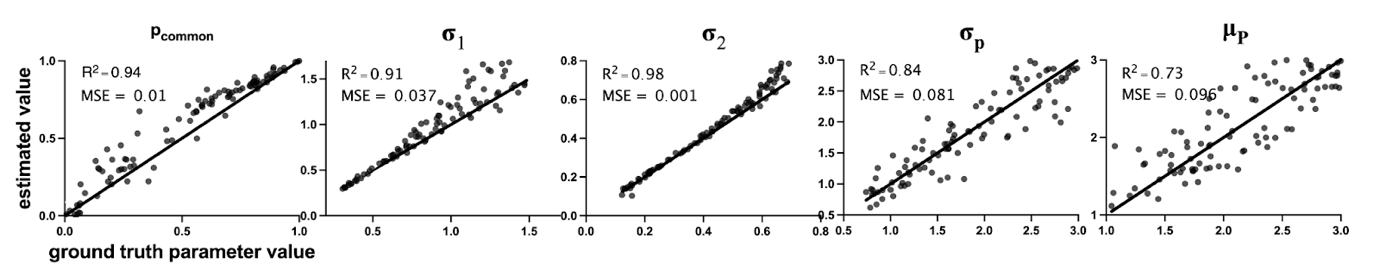


**Figure S3. Parameter-recovery accuracy for the two-dimensional BCI model.** Each panel plots the value recovered by the fitting procedure (vertical axis) against the ground-truth value used to generate the synthetic data set (horizontal axis) for one of the five free parameters: causal prior p_common_​, sensory noises σ_1_ and σ_2_ (visual and auditory numerosity), prior width σ_p_​, and prior mean μ_p_. Points represent 100 independent simulations; the solid line is the identity line (perfect recovery). The coefficient of determination (R^2^) and mean-squared error (MSE) for each parameter are printed inside the corresponding panel. Slopes near unity and high R^2^ values indicate that all parameters are identifiable with minimal bias.

**Model Identifiability and Discriminability Analysis**

To confirm that the one-dimensional (1D-BCI) and two-dimensional (2D-BCI) models are statistically distinguishable and that the fitting procedure can reliably recover the true generative model, we conducted a model identifiability and discriminability analysis using synthetic data. Specifically, we generated two synthetic datasets: one from the 1D-BCI model (which considers numerosity alone) and another from the 2D-BCI model (which jointly considers numerosity and temporal information). Each simulated dataset was then fitted with both models using identical fitting procedures and parameter initialization.

As shown in Figure S4, the model recovery results demonstrate excellent separability between the two models. When data were generated from the 1D-BCI model, the same model yielded substantially higher likelihood (lower –log likelihood) compared to the 2D-BCI fit (Panel A). Conversely, when data were generated from the 2D-BCI model, the 2D fit clearly outperformed the 1D fit (Panel B). These results confirm that the two models are distinguishable and that the fitting procedure reliably identifies the true underlying generative process.

**
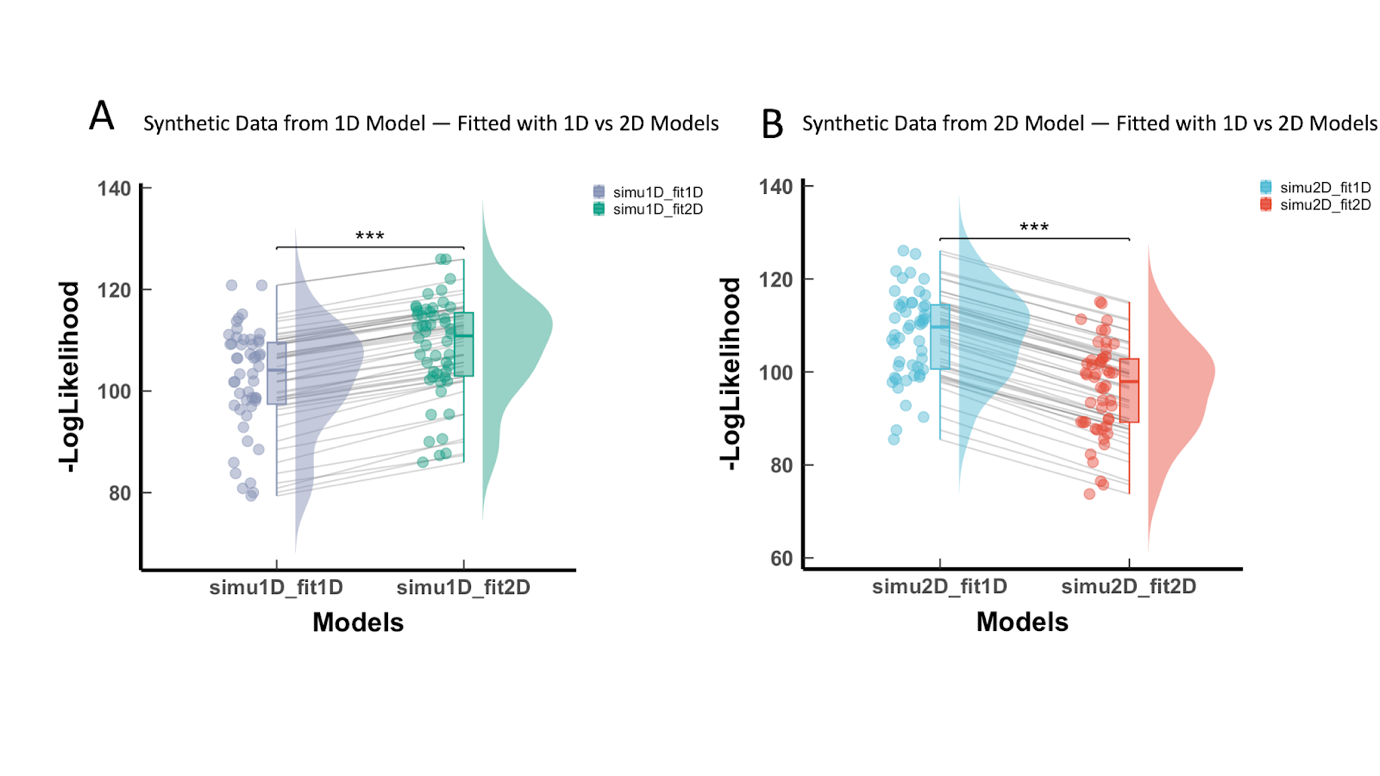
**

**Figure S4. Model recovery analysis for 1D and 2D Bayesian Causal Inference models.**(A) Synthetic data were generated using the 1D-BCI model and fitted with both the 1D and 2D models. (B) Synthetic data were generated using the 2D-BCI model and fitted with both models. Each dot represents one simulated participant; gray lines indicate within-simulation comparisons. Violin and boxplots show group-level distributions of the negative log-likelihood (–LL). Lower –LL values indicate better fits. Across both analyses, each model best recovered its own simulated data (***p < .001, paired t-tests), confirming the distinct identifiability of the two models.

**Model Parameters and Notation**

| **Symbol** | **Description** |
| --- | --- |
| s_A_, s_V_ | True auditory and visual numerosities (physical stimulus values). |
| x_A_, x_V_ | Noisy sensory measurements of auditory and visual numerosities. |
| t_A_, t_V_ | Observed auditory and visual onset times. |
| s_At_, s_Vt_ | True onset times of auditory and visual signals. |
| σ_V_, σ_A_ | Standard deviations of auditory and visual sensory noise for numerosity. |
| σ_Vt_, σ_At_ | Standard deviations of auditory and visual temporal noise. |
| μ_p_​, σ_p_​ | Mean and standard deviation of the numerosity prior. |
| μ_tp_​, σ_tp_ | Mean and standard deviation of the temporal prior (shared across modalities). |
| C | Latent causal variable (1 = common cause; 2 = independent causes). |
| P(C=1), *p_common_* | Causal prior: probability that the two signals arise from a common cause. |
| s_(A,C)_ , s_(V,C)_ | Posterior estimates of auditory and visual sources under causal structure (C). |
| *ϕ* | Latent temporal variable representing the true event time. |
| L | Log-likelihood of the observed data given model parameters. |
| BIC | Bayesian Information Criterion used for model comparison. |
